# Drake’s rule as a consequence of approaching channel capacity

**DOI:** 10.1007/s00114-014-1235-6

**Published:** 2014-09-17

**Authors:** Alexey A. Shadrin, Dmitri V. Parkhomchuk

**Affiliations:** 1 Fachbereich Mathematik und Informatik, Freie Universität Berlin, Berlin, Germany; 2 Department of Vertebrate Genomics, Max Planck Institute for Molecular Genetics, Berlin, Germany; 3 Apeary GMBH, Berlin, Germany

**Keywords:** Drake’s rule, Molecular evolution, Information theory, Neutral theory

## Abstract

How mutations accumulate in genomes is the central question of molecular evolution theories. However, our understanding of this process is far from complete. Drake’s rule is a notoriously universal property of genomes from microbes to mammals—the number of (functional) mutations per-genome per-generation is approximately constant within a phylum, despite the orders of magnitude differences in genome sizes and diverse populations’ properties. So far, there is no concise explanation for this phenomenon. A formal model for the storage of genetic information suggests that a genome of any species operates near its maximum informational storage capacity, and the mutation rate per-genome per-generation is near its upper limit, providing a simple explanation for the rule with minimal assumptions.

## Introduction

For about a hundred years, the key parameter in modeling Darwinian selection has been “fitness”—it defines which organisms survive and reproduce in a population and which are eliminated. Alleles or mutations (“variants”) are presumed to have different fitness, and the dynamics of variant destiny (its frequency in a population) is traced by a mathematical model.

There are numerous models with different assumptions about how to model real populations “correctly”. For example, the “Moran process” (Moran 1962) represents a model with “overlapping generations” where elementary time step is defined as a death of an arbitrarily chosen individual and the reproduction of another random individual, deriving the analytical solutions for some simple scenarios. Alternatively, the “Wright-Fisher model” (Durrett 2008) presumes the non-overlapping generations, such as annual plants. Then there are questions of how to calculate the cumulative fitness for a few independent variants, taking into account the effects of newly appearing variants, and many other subtleties. In the traditional models, the fitness is “relative”, i.e., it is normalized so it is distributed around the unit. In the case of calculating cumulative fitness for multiple alleles, sometimes formulas analogous to what we introduce here are used (Ofria et al. 2008; Strelioff et al. 2010; Frank 2012). However, in these cases, the absolute value is not interpreted and can also be normalized. For example, in traditional approaches, there is no sense including into fitness formulas sites that are not variable in a population (i.e., sites where any variation is lethal), and usually most of the genome is not variable in realistic modeling, while in our formula it is essential to sum overall sites to gain proper interpretation of fitness. Hence, for relative fitness, there is no fixed “baseline”—an individual cannot be assigned a fitness value ignoring the rest of the population. It is impossible to compare a fitness of an elephant to a fitness of yeast. Such fitness keeps no population history—a gain (or loss) of fitness for a whole population is untraceable, because after the gain the organisms are competing with each other formally in the same way. So the progressive evolution is presented as an opportunistic non-directional “Brownian” motion, fixation of accidental “positive” mutations.

It would be tempting to have a measure, which is “absolute”, has a baseline, and reflects the genetic complexity—the total “genetic information” or “evolutionary progress”. On one hand, this measure would allow us to compare different species. On the other hand, what is more important, this measure would be a natural choice for the fitness function within a population for modeling purposes. The proposed model is capable of recapitulating all traditional dynamics (e.g., “fixation”, “drift”, etc.); however, it quantifies an additional dimension—total genetic complexity. Modeling evolution without tracing this value can easily lead to “un-physical” solutions—when the complexity is allowed to wander arbitrarily in the course of sequence evolution. From common observations, it is natural to expect that a given species’ genomic complexity is a sufficiently preserved value on an evolutionary scale, despite the numerous ongoing changes in underlying genomic sequences. Then, if the complexity has changed significantly in the course of simulation, we should count the end product as a different species. Modeling speciation events per se is a very different subject from modeling species preservation. In essence, we model the very basic biological phenomenon, the preservation of form, while the matter (e.g., cells) in this form is continuously renewed. Instead of the matter, we show how functional genomics sequences can be continuously renewed while preserving species-specific phenotype, so the phenotype (typical set) and hence the total genomic information are invariants.

Besides introducing the invariant, the total genomic information, the “physical” property of our approach can be further illustrated by the stability notion: in order for a system to be stable under external perturbations (random mutagenesis can be considered as such) it must reside in a “potential well”. These perturbations are compensated for by forces that return the system to initial conditions. Without such compensations, the system would “smear out” in Brownian fashion. Random mutations can both increase and decrease genetic information (*GI*), and in our case, these compensating forces are selection, which tries to increase *GI*, and the channel capacity limit, which makes it impossible to maintain *GI* above a certain value and increasingly costly to approach the limit from below.

Despite the abundance of evolutionary models, their explanatory power remains arguably limited, so much so that in 1996 Ohta and Gillespie admitted “a looming crisis”: “all current theoretical models suffer either from assumptions that are not quite realistic or from an inability to account readily for all phenomena” (Ohta and Gillespie 1996). It is likely that the limits of the current models are rooted in the basic fitness definition and/or the absence of suitable genomic information measure, because if it is similar in all models the reshuffling of other parameters will not drastically change the behavior and predictions on a fundamental level.

Recently, we proposed an information-theoretical model (Shadrin et al. 2013) that can provide an “absolute” measure that estimates the total genomic information and that can be used for the fitness calculations, sensibly accounting for interactions of any number of variants in a genome. Technically, our fitness is quantifying the degree of “typicality”, and the size of corresponding “typical set” is related to genomic information or complexity. Fitness connection with complexity is the most essential difference of our model from the traditional approaches, while the modes of reproduction and other parameters are of secondary importance. Due to the novelty of such (interpretation of) the fitness function, we have to explore the model properties starting from very basic considerations, omitting the moment phenomena which are routinely considered in standard models, such as the influences of recombination, linkage, sexual selection, fluctuating environment, and so on. Though clearly, such phenomena would be interesting to include in the subsequent development of the model and to compare results with traditional approaches.

Technically, our model can accommodate any fitness expression including “relative” variations. However, in the latter case, additional care should be taken to monitor the equilibrium condition and the complexity dynamics, while the suggested “absolute” fitness expression automatically makes these tasks trivial.

An interesting approach for quantifying complexity and modeling its increase for digital organisms was suggested in Adami et al. (2000). Our approach is different—we quantify information by a mechanistic model of molecular interactions and are mostly concerned with the preservation of such information with mutation/selection balance.

The so-called Drake’s rule (Drake 1991; Drake et al. 1998; Sung et al. 2012) is an observation that states: within broad groups of organisms, the density of accumulated mutations per generation is roughly inversely proportional to genome size, which can vary by a few orders of magnitude. Naturally, the genome size and total genomic information are somehow related. While this relation can be complex in general, it is clear that accounting for this information might shed light on the coevolution of the mutation rate and genome size, and hence on Drake’s rule.

Another closely related phenomenon is the “molecular clock”—the rate of mutation accumulation is roughly proportional to the time of divergence from the last common ancestor. Roughly speaking, the “molecular clock” is a manifestation of Drake’s rule on the evolutionary timescale, because despite the fluctuations in genome sizes and numerous population properties in the course of divergence (assuming the generation time changing slowly), the clock is sufficiently monotonic when comparing gene sequences (i.e., mutations density and rate are constant). Usually, it is explained with the neutral theory—the majority of mutations are behaving “as if” they were neutral (Kimura 1983). However, it is not clear how to properly apply the neutral theory to the molecular clock—the clock is ticking monotonously but at a different pace in strongly and weakly conserved genes. Should we then introduce some kind of differential “neutrality density” in order to accommodate “neutrality” for the explanation? A cleaner approach is to treat mutations with a continuous spectrum of effects—from zero to lethal (fully conserved position). In this case, it is clear that point zero (pure neutrality) is not special, because the next infinitesimally close value (minuscule functional) will have the same properties. It seems that the aim of the neutral Theory is not the point zero per se, but some loosely defined region in its vicinity.

At the time of its appearance, due to the absence of computers, the neutrality assumption served as a useful simplification explaining a number of phenomena as it postulated that stochastic processes without selection drive the majority of mutations. However, consider a mutation in a “non-functional” genomic region—first it will change replication dynamics due to different weights, shapes, and abundances of nucleotides, then it will affect the local chromosome or chromatin shape. These changes can be minuscule; however, in a strict mathematical sense the resulting organism is different. Hence, the strict neutrality is an exception rather than a rule. Now, we do not need the simplifying assumption for computer modeling—all mutations can be counted as functional with arbitrarily small effects if required. Here, we show that while (expectedly) most genomic dynamics can be attributed to the mutations with small effects, the less recognized issue is that their cumulative contribution to genomic complexity evolution can be significant due to their abundance. There are experimental indications of this phenomenon (Yuan et al. 2013). Indeed, collectively they can behave “as if” they were neutral, however, the reason for this is not their weak functionality per se (we show that the degree of functionality does not matter), but the “saturation” of genomes with information, the proximity to the “channel capacity”.

Random mutations deteriorate the genomic information and must be compensated for by selection to maintain the total genomic information. Here, we illustrate some simple scenarios of such a process under equilibrium condition. With some arguably plausible assumptions, such a process readily explains Drake’s rule and molecular clock without involving neutrality. Here, we address the purely theoretical (postulated) phenomenon of Drake’s rule, while its experimental validity for all species is quite a different subject and not covered here. In fact, the provided theory may suggest some clues about species which are the “outliers” for the rule, having a significant deviation from the trend.

## Information in sequence patterns

The measure of genetic information (*GI*) was proposed by Schneider et al. (1986). It represents an adaptation of the entropy concept from the Shannon’s information theory (IT) (Shannon 1948) to the context of biological sequences. During the last 25 years, it became a popular tool for the investigation of variability of functional sequences (Schneider and Stephens 1990; Hertz and Stormo 1999).

The acceptable variability in each position (*P*) is defined by the frequencies of four nucleotides in an equilibrium population and quantified by Shannon’s entropy:

1 $$ H(P)=-{\displaystyle \sum_{N\in \left\{A,G,C,T\right\}}{f}_N{ \log}_2{f}_N} $$

where *f*_*B*_, *B*∈{*A*,*G*,*C*,*T*} is the frequency of nucleotide *B* at the position *P*. The genetic information for a single position is defined as *GI*(*P*) = 2-*H*(*P*). One possible interpretation is that such function conveniently (additively and linearly) quantifies the amount of biases from the uniformity in equilibrium distribution of alleles.

For technical simplicity, we (after Schneider et al.) assume independent positions in patterns (no epistasis), otherwise we would have to deal with general “typical sets” and the *GI* computation would be more complicated. However, there are no indications that assuming some positional dependencies in patterns would drastically influence the main conclusions. While covariable sites are known, significantly correlated sites can be grouped in “pseudo-sites” (now with more than four states) so that correlations can be canceled (concealed) with a proper basis selection. For example, RNA viruses may have numerous secondary/tertiary structures and thus many strongly covariable sites, so the above assumption is violated. However, such genomes still have some total genomic information, which is more difficult to calculate formally. Our simplified calculations here merely illustrate the general principles: the interplay of the total genomic information, genome size, and mutation rate. While complexities of formal *GI* calculations in diverse-specific cases can be interesting to investigate, the basic principles of genomic complexity evolution, which we discuss here, are invariant.

Recently, we showed (Shadrin et al. 2013) that the sum of *GIs* can serve as a measure of localization information. This “additivity” should not be confused with a simple additivity of Shannon’s entropy—the problem is to prove that the sum of *GIs* for a functional sequence (or a genome) is linearly linked to the “localization information” (the specificity of molecular interactions), i.e., the information required to locate a sequence in the corresponding sequence context. One could use other measures of frequencies bias—why is the defined one “fundamental”? In order to sum up the positional *GIs* to discern the informational meaning, the number of possible functional variants for the sequence (the size of its typical set) must depend exponentially on the defined variability of the sequence (the value reciprocal to the sum of *GIs*). This exponential dependence is the non-trivial result of the IT (Shannon–McMillan–Breiman theorem). The corresponding “natural choice” of the logarithmic function for information measure is discussed in detail in the classical Shannon paper (Shannon 1948). With such a well-defined positional information measure, it is possible to build a formal (“mechanistic”) model for the evolution of “molecular machines”.

Then we can use the position-specific *GIs* to calculate the total amount of information contained in a population of genomes as a simple sum over all positions:

2 $$ G{I}_{total}=2L+{\displaystyle \sum_{j=1}^L{\displaystyle \sum_{B\in \left\{A,G,C,T\right\}}{f}_{iB}{ \log}_2{f}_{iB}}} $$

where *f*_*jB*_, 1 ≤ *j* ≤ *L*, *B* ∈ {*A*,*G*,*C*,*T*} is the frequency of nucleotide *B* at the position *j*. Let us also define an average density of genetic information in a population as *GI*_*ρ*_ = *GI*_*total*_/*L* bits per sequence. It is obvious that 0 ≤ *GI*_*ρ*_ ≤ 2.

Hence, a functional sequence (or a genome) is represented by the corresponding pattern, the “*GI* profile”, so that *GI* and the corresponding 4-vectors of the acceptable equilibrium frequencies are defined in each position. As we discussed in Shadrin et al. (2013), the equilibrium condition is important for the correct *GI* definition and measurement, while, in general, real populations are far from the equilibrium. Importantly, the *GI* profile is the “prior”, inherent property of molecular functionality, for example a protein domain can be functional only within a certain set of sequences, e.g., in *GI* terms, and a conserved domain has high *GI* value and a small “typical set”. So, we posit that a given species is fully characterized by the set of all possible sequences, which produce the species-specific phenotype and define the typical set. It is clear that this set is much smaller than all possible random sequences. However, in general, it is much larger than a realistic population size. For this reason, we need to simulate the equilibrium in order to enumerate the complete typical set. Then, the average density *GI*_*ρ*_ cannot be significantly different in close species—functional genes are conserved similarly, unless some novel mechanisms of molecular functioning are introduced. The actual variability in a population depends on this predefined *GI* and a population history. The equilibrium population, which we simulate here (effectively canceling out, “erasing” the history influence, and revealing the unobscured “pure functionality” profile), is necessary for the correct *GI* measurement (the knowledge of the complete typical set) and the determination of an “error threshold”. However, a slice (a small subset) of such a population will have the same mutational properties as the whole equilibrium population, but smaller variability. Such subset represents a realistic population, which recently (relatively to mutation rate) underwent a bottleneck and experienced a “founder effect”, that is, all individuals are closely related through a few recent population founders. The variability in this subset does not correctly reflect the *GI* profile. However, this profile still “exists”, though more in a platonic sense. It could be revealed if this subset was allowed to diverge for a sufficiently long time without any disruptive events. This equilibrium population shows the principle limit on the maintainable pattern (revealing the full typical set, total *GI*, quantifying the total amount of biases), which is then defined solely by the mutation rate and reproduction/selection properties of the population, since the dynamical part (“history”) is excluded. It is clear that, with other things being equal, this limit plays the same limiting role for the “collapsed” population (after a bottleneck). We can imagine that under the influence of mutagenesis a realistic population is drifting inside a large typical set. Nonetheless, this is much more restrained drift in comparison with the drifting in a space of all possible sequences by random walk. However, since a typical set can be huge, in general, the drift might give an impression of a random walk.

Such modes of mutagenesis and maintenance of variability are similar to those in quasispecies theories: “The quasispecies concept becomes important whenever mutation rates are high. This is often the case in viral and bacterial populations” (Nowak 1992). In these theories, a population is represented by a “cloud” of diverged genotypes. However, the distinction between “normal” species and quasispecies is blurred, and nothing can prevent us from viewing a “normal” population as the aforementioned subset of a quasispecies (in the process of divergence). Here, we assume that this mode of high mutation rate is precisely that which deserves careful examination in the large genomes of higher organisms as well—what matters is the mutation rate per-genome per-generation, and as we now know, this parameter is quite large in mammals (and other highly evolved forms) at about a few hundred mutations, with few in coding regions. This is actually the main point of Drake’s rule.

For simplicity, we presume that selection has an opportunity to act in a compensatory manner (to increase *GI*) only in-between generations, ignoring possible germ-line selection issues. That is the reason for focusing on the per-genome per-generation mutation rates—selection does not “see” a genome size or per-base mutation rate. What it does “see” is the cumulative effect of all the mutations in the genome, which it tries to compensate through genetic deaths, the removal of the genomes from a population. So, the natural “units” for selection actions are a genome and a bunch of mutations in it. In comparison, the quasispecies theory is used to address the evolution of HIV with 1–10 mutations per division, so from the selection point of view the functional impact (at least in *GI* terms) is comparable. As pointed out by Nowak (1992), a given HIV population “seems to operate very close to its error threshold”. The existence of the “threshold” is our main postulate here. However, we apply it to all species, and with the provided IT framework, such a threshold seems to be well defined and ready for modeling. The main difference between virus and mammals populations seems to be the generation time and the genome size—the virus genotype “cloud” can be readily observed empirically. However, to generate the actual equilibrium “cloud” for a large, slowly replicating genome would take an astronomically large time and population size—equivalent to enumerating the full typical set. Nevertheless, this does not mean that we cannot explore the properties of this limit theoretically and then assume that these properties are applicable to the aforementioned population slice. The equilibrium mode of maintaining variability is considered in quasispecies theories too, and after we introduce the pattern definition and the measure of genetic information with fitness function, we arrive at our model. However, in the quasispecies theory, the fitness is defined for the whole population of mutants, not for individuals (Nowak 1992).

The analogy with quasispecies serves illustrative purposes but should be taken with a grain of salt—there are important differences from our model. For example, the quasispecies “cloud” arbitrarily depends on replication/mutation rates and other parameters, and has no deeper meaning, while our “cloud” represents the typical set as defined as all genotypes producing a species-specific phenotype (defining species total *GI*, which is missing in quasispecies model). Our threshold arises from the IT notion of channel capacity. Although it is conceptually similar to the “error threshold” from the quasispecies model (both, in essence, speculate about an inability to maintain species-specific phenotype) (Eigen 1971), they have substantial differences. While quasispecies model considers dynamics of infinite populations with non-lethal mutagenesis, our model allows for finite populations with lethal mutagenesis (which is the realistic scenario). Also, Eigen’s error threshold is not equivalent to channel capacity, in the sense that in the latter case there is no unavoidable “catastrophe”—information can persist with arbitrarily low sequence conservation, where information density can be arbitrarily low, as we discuss below. Analogously, in IT, reliable transmission is possible with any noise level, but the rate will be lower for higher noise.

Another related question is that the mutation rate must be sufficiently high to invoke the “quasispecies dynamic”, otherwise the expediency of the quasispecies approach can be challenged (Holmes and Moya 2002; Wilke and Adami 2003; Wilke et al. 2001). Some microbes (e.g., wild-type *Escherichia coli*) have mutation rates significantly lower than one per-generation, though they still acceptably fit into Drake’s rule because it holds on a logarithmic scale. In such a case, our simple simulation will converge to trivial monoclonal population, actually reflecting in vivo situation for a single bacterial colony. To regain Drake’s rule and molecular clock phenomena, we need then to revise some simplifying assumptions. A good candidate is constant environment—if the environment is oscillating, then we effectively have many different *GI*-profiles (or one multidimensional) where the global population is distributed and individuals are moving from one environment to the other. In this case, the effective (global) mutation rate must be higher, when averaging over all sub-environments and population transfers between them, and we will regain a “cloud” instead of monoclonal population. In lay terms, for a species in a fluctuating environment, it would be advantageous to have some “memory” about different and recurring sub-environments, instead of adapting to them de novo at every encounter. In that case, the decreased mutagenesis can provide this improved memory. For a single sub-environment, such species would look overly complex and the mutation rate would be below the Drake’s rule prediction. Lineages with higher mutation rate were wiped out by the environmental fluctuations.

These considerations lead to an interesting prediction: microbes enjoying more stable environments should have higher (properly normalized) mutation rates. This seems to be consistent with observations: wild-living microbes usually have lower mutation rates in comparison with parasitic relatives who enjoy a host’s homeostasis. Usually, the increased rate is attributed to the “arm-race” with a host’s immune system; however, the story might be more complicated—the arm-race (an increased mutagenesis) could be confined to a few specific genes (which are indeed observed in some cases, e.g., cell-surface proteins and so on) while the whole-genome elevated mutagenesis is costly and is not so well-motivated. When wild-living microbes “compete” with a significantly changing environment, it might impose the “racing” pressure genome-wide, because of large differences in entire metabolism in different environments.

In our model (for *GI*_*ρ*_ < 2 bit), a large number of allowable sequences (constituting a typical set) have nearly the same fitness and can coexist in a population in the case of equilibrium maintenance evolution. However, they are not completely equal in fitness so that selection can maintain a pattern by discarding the most deviant (“atypical”) sequences. Given the defined weight matrices of a desired conservation profile, the model provides selective values of individuals considering all mutations, present and de novo. Recently, we showed (Shadrin et al. 2013) that the substitution rate in functional sequences can be arbitrarily close to the neutral rate, and the fraction of positive mutation can be high in general. About 50 % of the retained mutations must be “positive”—a trivial requirement for the balance of *GI*.

This model, analogous to studying evolution with Turing machines (Feverati and Musso 2008), can be described as a population of machines operating on symbol sequences (of limited length), reading out positional information, recognizing corresponding patterns (via typical sets, technically, for a general typical set an assumption of positional independence is not necessary) of molecular interactions, and calculating a high-level phenotype. However, it seems that our machine is closer to describing the “molecular computations” through pattern recognition in comparison with the sequential algorithmic Turing machine and some other forms of digital organisms. For the purposes of this investigation, we do not have to specify the phenotype calculations per se—once we define the patterns and typical sets in a “genome”, we can address the problem of their maintenance or evolution (e.g., the cost or speed of patterns preservation or change). Here, we focus on the maintenance properties, treating such machines as genetic information storage devices that must resist the random noise of mutagenesis. The only computation is done for selection actions—a degree of genome “typicality” is used as fitness, accounting for all variants in a genome (Eq. 3). As could be expected, our fitness function is similar to the traditional one in its basic “common sense” features—for example, a mutation in a highly conserved site (high *GI*) will lower the fitness significantly. Notably, in this model, all sites and variants are functional—there is no need to postulate “neutral” (Kimura 1983) or (loosely specified) “near-neutral” (Ohta 1973) variants (to explain the high rates of sequence evolution). In our case, the equilibrium can be interpreted as the cumulative neutrality of all mutations (remaining in a population), while assuming the individual neutrality for the majority of mutations would be throwing the baby out with the bathwater.

## Simulation

### Simulation terms

An organism in the simulation is represented by the nucleotide sequence of given length (*L*), *O* = [*B*_*1*_, *B*_*2*_, …, *B*_*L*_], where ∀ *i*∈[1,*L*], *B*_*i*_∈{*A*,*G*,*C*,*T*}. A population is a set of organisms of the same length. The parameters that govern the simulation process are shown in Table 1. The mutational bias (of transitions/transversions) is included in the code for universality, but has no effect on the trends we investigate here. As we discussed in Shadrin et al. (2013), species-specific biases can play an interesting role for *GI* storage optimization and may slightly affect species dispersion along the Drake’s rule trend line. However, for brevity, here, we assumed it as constant.

**Table 1:** Simulation parameters

Notation	Description
*N*	Number of organisms in the population (population size).
*L*	Length (number of bases) of genome of each organism in the population.
*n*_*d*_	Number of descendants each organism produces in a single round of reproduction.
*P*_*m*_	Probability of mutation per base.
*P*_*ti*_	Probability that an occurred mutation will be a transition mutation.
*W* = (*W*_*j*_ | *j*∈[1,*L*])	Selection weights of nucleotides in each position, where *W*_*j*_ = (*w*_*jA*_, *w*_*jG*_, *w*_*jC*_, *w*_*jT*_), *W*_*j*_(*B*) = *w*_*jB*_, *B*∈{*A*,*G*,*C*,*T*}—selection weight of the corresponding nucleotide *B* in *j*-th position.

Each organism (*O*) in a population can be associated with a weight specified by the weight matrix *W*:

3 $$ W(O)=W\left(\left[{B}_1,{B}_2,\dots, {B}_L\right]\right)={\displaystyle \sum_{i=1}^L{W}_i\left({B}_i\right)} $$

A “typical” probability is the expected probability of a sequence for a given *GI*-profile. It can be estimated through multiplication of the expected frequencies for corresponding positions. Here, for computational convenience, we define the fitness as a sum of position-specific weights that, for our purposes, is equivalent to the multiplication thereof if we had used logarithms of frequencies. However, as we mentioned, any fitness expression (additive, multiplicative, etc.) for multiple alleles will produce some conserved pattern. Such (potentially interesting) complications can influence only the shape of the resulting *GI*-profile and its stability (fluctuations); they do not affect the existence of the mean density and the independence on the population size. However, for example, the specifics of reproductive success dependencies are obviously important for the dynamical part before the equilibrium is reached.

We do not know the resulting *GI*-profile before the simulation is performed. Hence, the weight matrix defines a general direction of pattern conservation by selection, not the actual *GI*-profile per se.

Weights (*W*) are used to determine selection preferences, which try to maintain a pattern. In our experience, the particular recipes for selection actions (e.g., probabilistic/deterministic) and reproduction modes (overlapping/non-overlapping generations) play a little role in the described trends, as long as the main purpose of these actions is to maintain a pattern, a biased frequencies distribution, while the opposing force, random mutagenesis, tries to flatten the bias. Each mutations round decreases the genomes “typicality”, on average. So a more “typical” genome has higher reproductive success, because its progeny is more likely to stay typical and avoid elimination. As we mentioned, *GI* can be viewed as a convenient measure of functionally acceptable variant frequency biases. Such an interpretation of fitness, which allows one to trace the total information value, is the key departure from traditional models. For example, it seems to be inherently difficult to approach the Drake’s rule explanation with a traditional fitness function, which is relative as it is “blind” to genetic complexity and genome size. In our case, the total *GI*, the genetic complexity, is measured by the amount of pattern (functionally acceptable) biases. It seems to be an intuitively appealing quantification: the larger the total amount of biases (further from the flat distribution), the higher the information content and energy required to maintain it. However, such an approach is a necessary simplification—it works under the assumption that the remaining (“higher order”) information unfolding processes are approximately the same, which should work at least for a similar species.

Presumably, the sophisticated error correction mechanisms, such as DNA repair, constitute a biological burden. Then, we can ask: what is the maximum mutation rate compatible with a given total *GI*? The differences of *GI*_*ρ*_ of functional sequences are assumed to be small for close species. Formally, for our phenotype-calculating machines, the conservation of *GI* is equivalent to the whole phenotype conservation, because, as we reasoned in Shadrin et al. (2013), conservation of *GI* preserves the positional information of molecular interactions, so that a phenotype is mechanistically derived from the whole genome pattern.

### Simulation process

The entire simulation process can be divided into three successive stages: initialization, spawning, and selection:

- *Initialization*: The initial population consisting of *N* organisms of length *L* is generated. All organisms in the initial population are identical and have maximum possible weight according to matrix *W*, i.e., at each position *j* of each organism stands a nucleotide *B*_*j*_: ${B}_j=\left[B\left|{w}_{jB}={\displaystyle \underset{B}{ \max }}\right.\left({w}_{jB},B\in \left\{A,G,C,T\right\}\right)\right]$.
- *Spawning*: The progeny is spawned. Each organism in the population produces *n*_*d*_ descendants (here we consider in detail only the case of binary fission, i.e., when *n*_*d*_ = 2). A descendant organism has the same length as its parent and is obtained by copying the parental sequence with a certain probability of mutation (*P*_*m*_) and with a bias of mutational spectrum (*P*_*ti*_). The parental organism is excluded from the population after the reproduction, so the generations are non-overlapping and after this step the population consists of *n*_*d*_ *N* organisms.
- *Selection*: Selection reduces the number of organisms in the population back to the initial size. It acts deterministically, leaving *N* organisms, whose weight *W*(*O*) is larger.

Initialization occurs only in the very beginning, and then the spawning and selection are repeated in a loop until the simulation process is stopped. The choice of procedure for generation of the initial population does not affect the steady state of the simulation process, so we can simply generate a random initial population. However, generating the initial population as described above will provide faster convergence to the steady state, the equilibrium condition, which reveals the “error threshold”, the goal of our experiments. The above mode of reproduction describes the non-overlapping generations for the simplicity of defining and counting mutations. However, we experimented with other modes, including the overlapping generations similarly to Moran model, and found the trends invariant.

## Results

### GI behavior in the course of simulation

Immediately after the initialization stage of the simulation, *GI*_*ρ*_ of the population according to formula (2) is equal to 2 bits because all organisms are identical. However, as we discussed earlier, this is not the “correct” functional *GI* (because a population is far from equilibrium), but a formally computed value in the course of simulation. If we start the simulation process as described above with the probability of mutation *P*_*m*_ high enough to allow occurring mutations to propagate in the population, then the diversity will emerge and *GI*_*ρ*_ will start to decrease. While reducing, *GI*_*ρ*_ will finally reach the level when mutagenesis is balanced by the force of selection and in consequent iterations will fluctuate in the vicinity of some value. The existence of the balance (mean *GI*_*ρ*_) is clear because the capacity (the averaged effect) of random mutagenesis to decrease *GI* monotonically drops from some value at *GI*_*ρ*_ = 2 to zero at *GI*_*ρ*_ = 0, while the corresponding selection capacity to increase *GI* behaves reciprocally by having non-zero value at *GI*_*ρ*_ = 0 and zero at *GI*_*ρ*_ = 2. Thus, these two functions intersect at some equilibrium point. In our numerical experiments, we consider that the population is already in the equilibrium state if during the last *T* generations (*T* = 100 in our tests) two conditions are met: The sum of all *GI*_*ρ*_ changes between consequent generations is less than a specified threshold (*1e-3* in our tests), the maximum number of consequent generations increasing/decreasing *GI*_*ρ*_ is less than *T*/10.

The observable magnitude of *GI*_*ρ*_ fluctuations around the equilibrium value depends on the size of the population, but the equilibrium value per se does not, which is natural to expect for the population maintaining constant (average) variant frequencies. So setting the size of the population (*N*) large enough, we can identify the moment of equilibration and equilibrium value of *GI*_*ρ*_ with the required precision. Even if we assume a more complicated scenario where the fluctuations are not settling down, the aforementioned capacities of mutagenesis and selection to change *GI* cannot depend significantly on the population size. They operate on the variants frequencies, which are disentangled from the absolute population size; hence the balance (even if it is the dynamic balance) between these two forces is also free from the population size dependence.

**Fig. 1:**
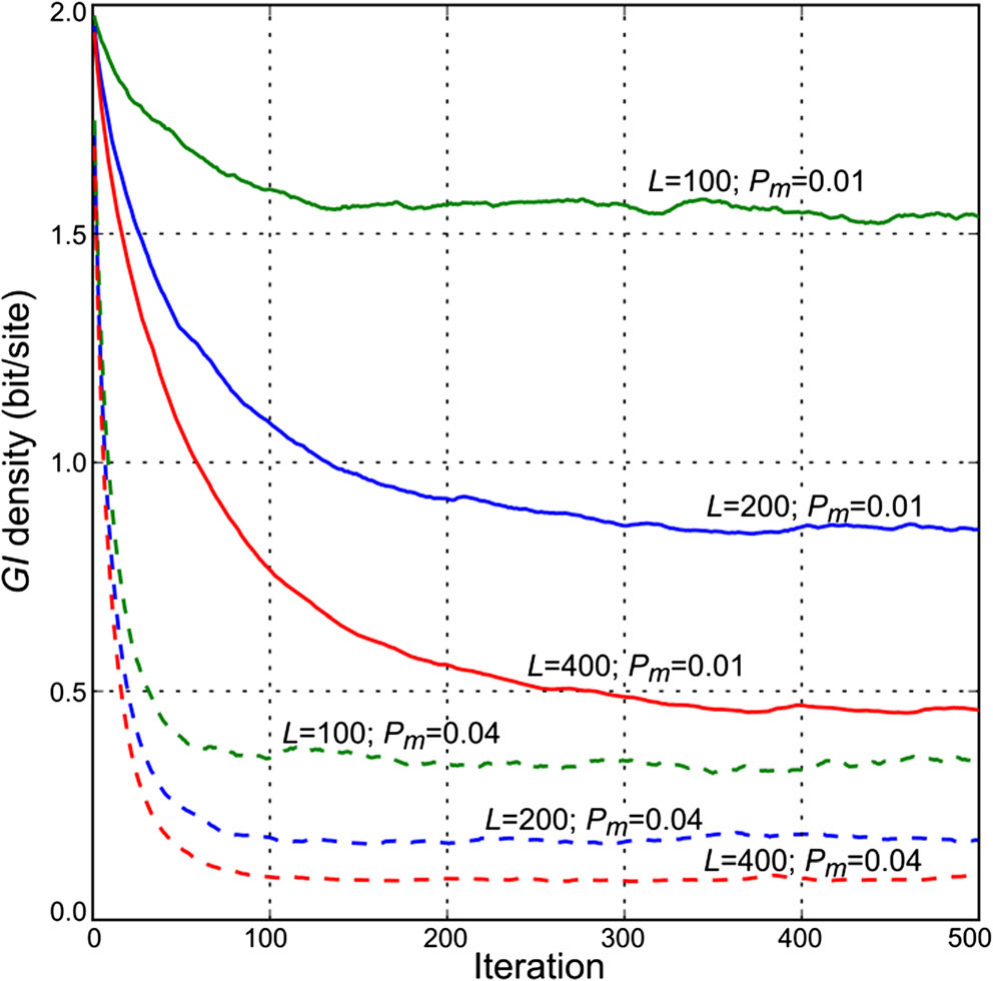
Convergence of *GI*_*ρ*_ for different parameters. Common parameters for all demonstrated cases are: *N* = 1,000; *n*_*d*_ = 2; *P*_*ti*_ = 2/3; *W* = (*W*_*j*_ = (0.8, 0.2, 0, 0) if j is even, else *W*_*j*_ = (0.5, 0.3, 0.1, 0.1)). Color determines organism length (*L*): *green* corresponds to *L* = 100, *blue* to *L* = 200, and *red* to *L* = 400. Line style determines probability of mutation per base (*P*_*m*_): *solid* corresponds to *P*_*m*_ = 0.01 and *dashed* corresponds to *P*_*m*_ = 0.04

**Fig. 2:**
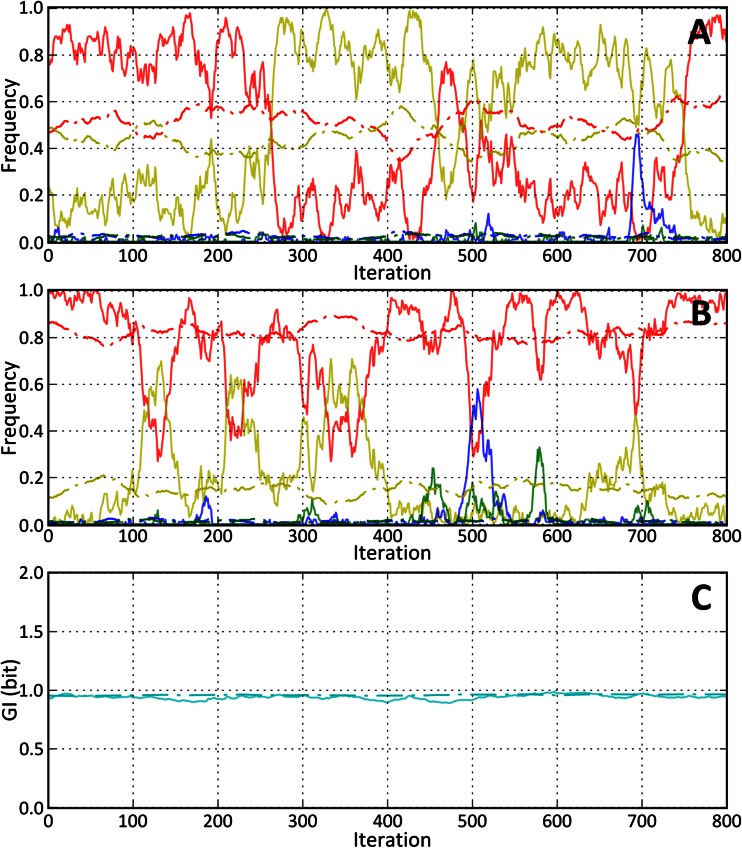
Fluctuation of positional nucleotide frequencies during *GI*-steady state for different selection weights (*W*) and population sizes (*N*). Common fixed parameters are *P*_*m*_ = 2^−6^, *P*_*ti*_ = 2/3, *L* = 128, *n*_*d*_ = 2. In all three subfigures (**a**, **b**, **c**), the line style defines population size: the *dash* and *dot lines* correspond to *N* = 10,000; the *solid line* to *N* = 100. **a** Fluctuations of nucleotide frequencies in a position (*P*) with selection weights *W*_*P*_ = (0.4, 0.38, 0.12, 0.1). **b** Fluctuations of nucleotide frequencies in a position (*P*) with selection weights *W*_*P*_ = (0.5, 0.3, 0.1, 0.1). **c** Dynamics of *GI*_*steady*_

We will call the state of the simulation when the population has already reached equilibrium of the *GI*-steady state and denotes the mean value of *GI*_*ρ*_ in equilibrium population as *GI*_*steady*_. The convergence of *GI*_*ρ*_ for different parameters is presented in Fig. 1. A biological interpretation for this state that it is a given species maintainable *GI* value. It can be called a “mutation-selection balance”, however, it is clearly different from Fisher’s balance (Crow 1986), who considered a single site, where in our case the balance is due to the compensatory effects of multiple positive and negative mutations. Other authors considered a balance similar to ours when the frequency of positive mutations is high so that they cannot be easily brought to fixation as in one-by-one case (Sniegowski and Gerrish 2010; Desai and Fisher 2007). This is also different from our approach in a number of aspects—we are not concerned with the fixations at all, and we quantify the limit on genomic complexity—as we discussed earlier, without considerations for this limit, a formal modeling might easily result in “un-physical” solutions. It should be clearly understood that the word “steady” here concerns only the total genetic information (and hence the phenotype), the genomes in the population remain variable, because new mutations still appear with the steady rate (see Fig. 2). The “molecular clock” is ticking, and its empirical steadiness on the evolutionary scale is another indirect hint that the average *GI* density is a slowly varying parameter. For example, mutations are more frequent in a position with lower *GI* value, so if density fluctuates strongly on the evolutionary scale, the clock would behave erratically. As we argued (Shadrin et al. 2013), *GI* increasing (positive) mutations constitute a significant fraction of random mutations (especially when *GI* in a position is low), thus allowing the same fraction (in the *GI* equivalent) of negative mutations to remain in the population. The monotonous molecular clock is a simple prediction of the provided model. Alternatively, it can be explained by the neutrality assumption, which seems to be an oversimplification of reality. Also, the provided model shows that the steadiness of the clock is intimately connected with Drake’s rule and the “error threshold”, while the neutral theory is inherently unable to make such connections.

### Counting mutations

In the simulation, the number of fixed mutations, i.e., the observed mutations per-generation can be counted directly. Following the common notation, we denote the number of mutations per-generation per base as *u*_*b*_ and the mutation rate per-generation per-genome as *u*_*g*_. Despite the fact that the values *u*_*b*_ and *P*_*m*_ are closely related, *u*_*b*_ is always less or equal than *P*_*m*_, since organisms with more mutations are more likely to be eliminated at the selection stage.

Now, let us look at the somewhat inverse experiment: we can fix the value of *GI*_*steady*_ and all parameters from Table 1 except *P*_*m*_, and then numerically find the value of *P*_*m*_ which corresponds to the fixed parameters. This procedure was performed for all combinations of different organism lengths *L*∈{64, 128, 256, 512, 1024}, different values of *GI*_*steady*_∈{1.2, 1.4, 1.6}, and different weights *W*∈{[*W*_*j*_ = (0.8, 0.2, 0, 0) if j is even, else *W*_*j*_ = (0.5, 0.3, 0.1, 0.1)], [*W*_*j*_ = (0.9, 0.1, 0, 0) if j is even, else *W*_*j*_ = (0.4, 0.3, 0.2, 0.1)]}. Other parameters in all experiments were fixed: *N* = 1,000; *n*_*d*_ = 2; and *P*_*ti*_ = 2/3.

**Fig. 3:**
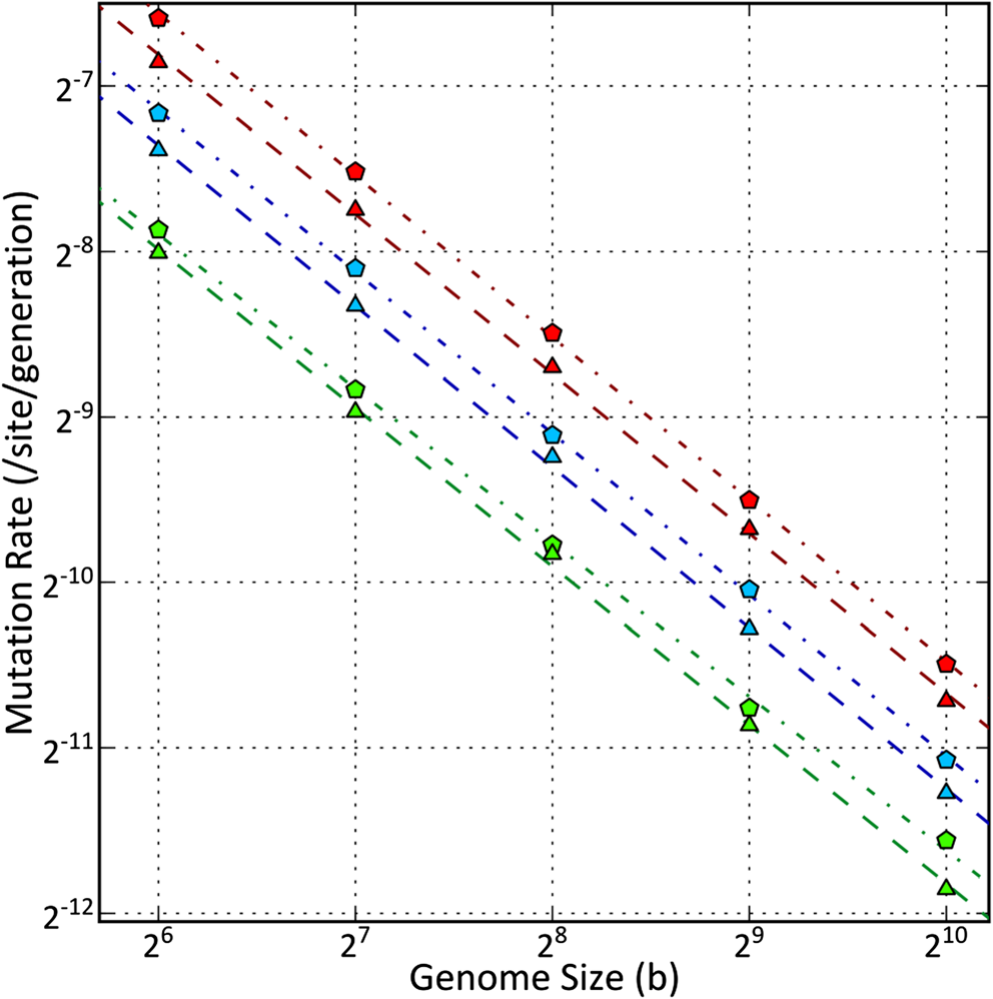
Relationship between the mutation rate per site per-generation (*u*_*b*_) and the genome size (*L*) observed in the simulation. Color determines density of genetic information in the steady state (*GI*_*steady*_): *red* corresponds to *GI*_*steady*_ = 1.2 bit/site, *blue* to *GI*_*steady*_ = 1.4 bit/site, and *green* to *GI*_*steady*_ = 1.6 bit/site. The shape of the marker determines selection weights (*W*): the *pentagon* corresponds to *W*_*pentagon*_ = (*W*_*j*_ = (0.8, 0.2, 0, 0) if j is even, else *W*_*j*_ = (0.5, 0.3, 0.1, 0.1)), the *triangle* corresponds to *W*_*triangle*_ = (*W*_j_ = (0.9, 0.1, 0, 0) if j is even, else *W*_*j*_ = (0.4, 0.3, 0.2, 0.1)). Lines represent linear regression on a log-log scale. *Dark red/blue/green lines* correspond to light red/blue/green markers; *dash* and *dot lines* correspond to pentagons, *dashed lines* correspond to triangles. Regression lines and corresponding correlation coefficients (r2): *GI*_*steady*_ = 1.2, *W*_*pentagon*_ (*red dash* and *dot line*): log_2_ *u*_*b*_ = − 0.68 − 0.98 log_2_ *L*, (r2 = 0.99). *GI*_*steady*_ = 1.2, *W*_*triangle*_ (*red dashed line*): log_2_ *u*_*b*_ = − 1.02 − 0.97 log_2_ *L*, (r2 = 0.99). *GI*_*steady*_ = 1.4, *W*_*pentagon*_ (*blue dash* and *dot line*): log_2_ *u*_*b*_ = − 1.29 − 0.98 log_2_ *L*, (r2 = 0.99). *GI*_*steady*_ = 1.4, *W*_*triangle*_ (*blue dashed line*): log_2_ *u*_*b*_ = − 1.52 − 0.97 log_2_ *L*, (r2 = 0.99). *GI*_*steady*_ = 1.6, *W*_*pentagon*_ (*green dash* and *dot line*): log_2_ *u*_*b*_ = − 2.31 − 0.93 log_2_ *L*, (r2 = 0.99). *GI*_*steady*_ = 1.6, *W*_*triangle*_ (*green dashed line*): log_2_ *u*_*b*_ = − 2.23 − 0.96 log_2_ *L*, (r2 = 0.99)

In the experiments, we estimated the number of mutations observed in the *GI*-steady state and compared *u*_*b*_ parameters for different genome lengths. The results are summarized in Fig. 3.

**Fig. 4:**
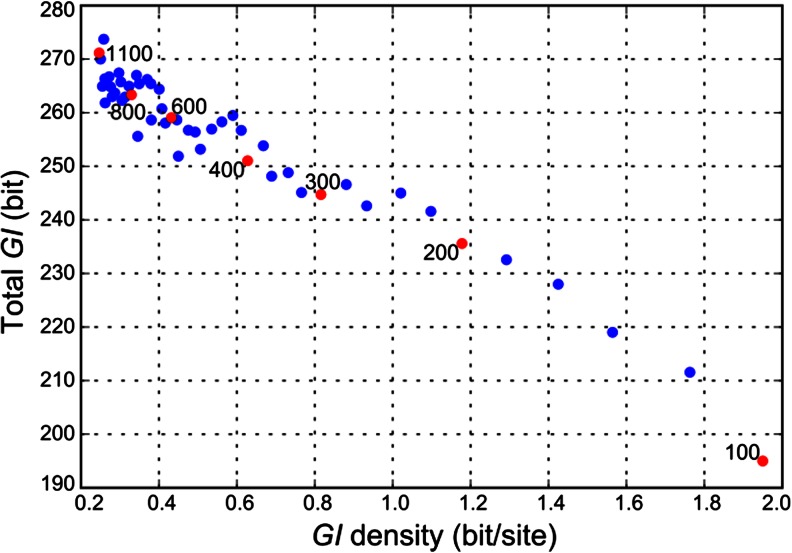
Dependence of total genetic information (*GI*_*total*_) and density of genetic information (*GI*_*ρ*_) on the length of genome (*L*) when the rate of mutations (*P*_*m*_) is fixed. Each point represents a population with organisms having genome of size *L*∈[100, 120, …, 1080, 1100]. For convenience of orientation, some points are colored in red and genome size of corresponding population is labeled. Mutation rate (*P*_*m*_) was fixed to 0.007. Also, all other parameters were identical for all populations, namely *N* = 1,000; *n*_*d*_ = 2; *P*_*ti*_ = 2/3; *W* = (*W*_*j*_ = (0.8, 0.2, 0, 0) if j is even, else *W*_*j*_ = (0.5, 0.3, 0.1, 0.1))

Figure 4 shows total genetic information (*GI*_*total*_) and density of genetic information (*GI*_*ρ*_) depending on the genome length (*L*) when the rate of mutations (*Pm*) is fixed. For each population displayed *GI*_*ρ*_ and *GI*_*total*_ were averaged over 1,000 generations after the population reached *GI*-steady state.

Through defining different weight matrices (Eq. 3), we tested different scenarios of the density distribution: with homogeneous *GI* distribution in a genome and bimodal—one half of a genome consisting of highly conserved (“lethal”) sites—to model the regions such as conserved protein domains and the other half consisting of weakly conserved sites, to model the variable parts of proteins, and weakly conserved non-coding regulatory DNA. The behavior of *GI* density versus mutation rate holds similar in all modeled scenarios, so that the actual weight distribution does not affect described trends.

## Discussion

A simple simulation (with rather predictable results) was used merely to illustrate the working of general principles we introduced. More realistic implementations are easily conceivable through modifying and extending our simplifying assumptions. For example, particular alleles might interact in more complex ways (i.e., epistasis) than described above, and as we mentioned, such interaction can be accounted for by constructing more complex typical sets. However, these complications cannot influence the basic conclusions, which rest on general principles without dependence on specifics of implementation. Furthermore, since the model provides reasonable explanations for observable phenomena with a minimal number of parameters and assumptions, and is computationally realizable, operating similarly to our understanding of molecular interactions, we believe that the model fairly captures the general properties of real genetic systems. Interestingly, the model can be considered a simple generalization of Hardy–Weinberg equilibrium (HWE) (Hardy 2003), explicitly including functional sites and their maintenance selection. HWE states that purely neutral alleles (in the absence of selection and linkage influences) maintain their constant frequencies in the sufficiently large (to avoid sampling/drift effects) equilibrium population. However, our model states exactly the same for the equilibrium population, but for the functional alleles, which do produce differences in fitness and selection (of arbitrary strength)! This may explain the persistent (about half-century) illusion of the neutrality in common tests (e.g., Tajima’s D (Tajima 1989)). Mutations in equilibrium population will “pretend” to be neutral, so such criterions actually test for the (local in the case of recombining population) equilibrium condition, rather than for the individual mutations neutrality. Below, we discuss some possible consequences for our understanding of real genetic systems, assuming that the model is sufficiently valid.

Naturally, we do not reject possible roles of classical phenomena. For example, the “strong selection”, which leads to “selective sweep”, is a non-equilibrium event and falls out from the applicable domain of the equilibrium model. In terms of *GI*, such events alone provide only 2 bits of *GI* for a given site, for the price of the total population replacement (roughly speaking). Such events are equivalent to considering a changing environment. Under the model’s assumptions of the constant environment and the infinite time equilibrium, all such events would occur and settle down. However, even for the changing environment situation, we propose that the model describes the “background” of such (presumably relatively rare) events. The number of such events must be limited by Haldane-type arguments (Haldane 1957), so we assume that the rest of mutational background might be better described by the provided model, than by the neutral approximation. In fact, according to the model, a mutation per se, with any selective value, while changing individual organism’s typicality or fitness, cannot increase the amount of total *GI* in the equilibrium population—the phenomenon we explain below. Hence, in this work, the model has well defined restricted applicability domain. However, it is straightforward to extend it to certain non-equilibrium scenarios such as abrupt or gradual changes in *GI*-profile, simulating changing environment. Admittedly, most variants in real populations are of weak effect, however, their number can be quite large, so that their collective effects can be far from negligible (as in the simplistic interpretations of the neutral theory), and our theory suggests a consistent way of accounting for such effects.

According to Drake et al. (1998), the genomic mutation rate “is likely to be determined by deep general forces, perhaps by a balance between the usually deleterious effects of mutation and the physiological costs of further reducing mutation rates”. As can be seen, Drake correctly did not include considerations for adaptive properties of evolution, practically solving the problem, hinting rather that it is the maintenance-related phenomenon, and once we interpret the maintenance as the equilibrium in allele frequencies—the main property of our model—the population size is obviously out of the equation (as in the case of HWE).

The key assumption for Drake’s rule explanation is that the total genomic information is saturated to its maximum maintainable value, or reciprocally and equivalently, the mutation rate is near its upper limit for a given species total *GI*. The mutation rates and thus the total *GI* are assumed to change slowly on evolutionary time scale. We hypothesize that the rate decrease is a basic event required for progressive evolution, and it is promptly followed by the gain in total *GI*, restoring the equilibrium. The equilibrium can be regained “quickly” (∼100 generations, judging by the speed of convergence to the steady state in Fig. 1).

One difficult question is how to motivate the stability of the mutation rate for a given species. For the rate decrease, we can assume that it might happen due to the large difference of the time scale of two phenomena. The first is merely a long-term advantage of the lowered mutation rate: some generations must pass to fill newly accessible *GI* (if a niche requires it, which does not have to be the case, in general). The second is the immediate disadvantage: “physiological costs”—since the lower rate, in principle, must be associated with a slower replication rate and/or additional energy expenditures. On the other hand, why are the rates not degrading, if increasing the rate might yield a quick advantage and only a disadvantage in the long-term? At this point, we can only speculate that for higher organisms, an increased somatic mutagenesis might also create a short-term disadvantage, preventing the rate degradation (e.g., somatic mutations theories of aging or carcinogenesis). Beside the somatic mutagenesis, we could imagine any other selectively important phenotype somehow linked to the changes in mutation rate. The other idea is that while the rate decrease must come at some “physiological costs”, the way back is not that easy—a mutation, which degrades the rate, will not necessarily reduce the “physiological costs” back to the previous values. Such a mutation must be a rather specific “back-mutation” or, more likely, a number of them, making it improbable to achieve both the rate increase and the corresponding costs reduction. Hence, the rates can only go down, locked from above by both short- and long-term disadvantages. Alternatively, the rate maintenance might require a regular population renewal, described below. Naturally, there are examples of regressive evolution, which can be easily caused, for example, by moving to a simpler niche (habitat)—“use it or lose it”. Interestingly, for our model, regressive evolution is not a priory less frequent than progressive; given sufficient niche separation, a subpopulation with increased mutation rate will degrade to a simpler species. This can probably be subjected to experimental verification, one simple example of (organ-specific) decrease of complexity is the blind salamander living in caves, which has “atavism”: rudiments of the eyes (sometimes even a lack of eyes). Wider observations of such reversed “atavisms” might shed light on how popular “degrading” evolution is. Technically, there are no reasons for rejecting the possibility that a number of simpler forms might “devolve” from more complex ones. While the latter must have ascended from some simpler ancestors, in principle, the topology of the evolutionary tree might resemble a willow tree (i.e., numerous descending branches, from a few thick nearly horizontal branches—“living fossils”).

Hypothetically, a change in mutation rate would remold a species phenotype (suggesting an explanation for “punctuated equilibrium” phenomenon), since small relative changes in the mutation rate can provide a significant absolute change in total accessible *GI* and a correspondingly significant change in the phenotype. In principle, it is assessable experimentally: if we were able to select flies (for example) for a lowered mutation rate, then the model predicts that such a population has the capacity to produce a more advanced species of flies. The problem is, however, that the population must be challenged with the proper external conditions, which could cause evolutionary progress, thus promoting an increase of complexity.

It is natural to expect that the rates occupy discreet values, due to the discreet nature of corresponding modifying mutations and their (presumably) limited number. We could also hypothesize about speciation scenarios: suppose that in a large population the rates are heterogeneous and mixed, so that the population has some average rate. Then after a “founder” splits off, he produces a new population that has a potentially different rate than the main population, leading to the fast phenotype changes.

The evolution of the (functional) genome size is presumed to occur through gene duplications (Ohno 1970), so that “gene families” grow in size. That also motivates our postulate of slow changes in *GI*_*ρ*_ for functional sequences—new sequences perform molecular functions similar to the original. The provided theory readily predicts that whole or partial genome duplications would lead to an increased rate of sequence evolution and to a subsequent shrinking back of the (functional) genome size, losing extra gene copies, due to inability to maintain higher total *GI* without changing the mutation rates. Evolutionary progress (an increase in complexity and *GI*) is happening not due to duplications per se (which are relatively frequent events), but due to the mutation rate decrease and/or the adoption of the lower *GI*_*ρ*_ functionality (Fig. 4)—these changes are assumed to be “slow”. This hypothesis can be supported by several recent experimental studies of RNA viruses. It is known that large- and intermediate-sized nidoviruses encode an enzyme implicated in controlling RNA replication fidelity, while other single stranded RNA viruses, with smaller genomes, do not encode the enzyme (Lauber et al. 2013). Nga et al. (2011) argued that an acquisition of this enzyme might have promoted genome extension. From the other side, Eckerle et al. (2007) demonstrated that viruses containing a defective mutant of the enzyme-encoding gene possess an enhanced mutation accumulation rate. However, as we mentioned, progressive evolution naturally intervened with external conditions (niche or habitat) and must be sufficiently complex to support the increase of species complexity. Duplications might also cause a reproductive isolation. Hence, together with the founder-specific mutation rate hypothesis, this might be a path to speciation and progressive evolution (when the founder retains the lowered mutation rate).

We suggest that the “channel capacity” notion of IT is sufficiently deep and can serve as a general principle to provide the desired understanding of Drake’s rule. The notion also allows for a quantitative modeling of the process. Channel capacity is the upper bound on the information transmission rate for a given noise level. Practical solutions for information transmission are somewhat below this theoretical limit, and considerable engineering efforts are dedicated to approach the limit, simply because being closer to the limit saves energy. Hence, yielding another basic consideration: if nature does not use the genomic informational capacity to its full extent, it would not be “thrifty”—why waste resources on the unused capacity? Thriftiness should be favored by selection (though there are some opposing ideas of “selfish” or “parasitic” sequences). If we presume that the early genetic systems operated at the “error threshold”, it is not clear at which moment and for what reasons this threshold was abandoned. It seems to be the thriftiest and the fastest way to progress, to stay always on the threshold, which is moving up due to the enhancements in replication fidelity and other possible mechanisms. In fact, considering the “costs”, it is difficult to come up even with an artificial reason to push fidelity beyond necessity. Thus, unless we discover some good motives for this reason, we have to admit (following Occam’s lines) that contemporary species are also at the “error threshold”. It seems that ignoring this fundamental threshold would make the evolutionary modeling critically incomplete.

Intriguingly, in IT, the problem of approaching the channel capacity limit has no general solution applicable to all practical situations, as it relates to the problem of achieving the best compression rate, and in practice, is limited by the memory and computational costs. That creates a recursive, self-referenced evolutionary system: in order to become more effective, resources must be invested in some analogs of memory and computations, and these resources, in turn, must be used in the thriftiest way, i.e., optimizing the optimization and so on, producing a Gödel-like system. Analogously to Chaitin’s proposals (Chaitin 2012), we can speculate that molecular machines have an infinite field for exercising the mathematical creativity in attempts to approach the limit, explaining the drive to complexity in living systems (Shadrin et al. 2013). The physical restraints (e.g., energy conservation) are thus the necessary prerequisites for forcing beings to explore the “Platonic mathematical world” (Penrose 2005), while the “Mental world” might arise out of necessity for memory and computing. Naturally, the simple model captures only the general properties of genetic information processing as there are many features not included—epigenetics, rearrangements, roles of transposable and repetitive elements, recombination, multiple ploidy, and so on.

In large genomes, there is a large number of repetitive and transposable elements (sometimes called “junk DNA”), seemingly challenging the thriftiness notion. However, we showed (Fig. 4) that in case of informational saturation it might be advantageous to utilize a low-density *GI* strategy (e.g., the ENCODE project (The ENCODE Project Consortium 2012) seems to support the broad functionality of intergenic regions). From the IT point of view (semantically), if we had a hundred copies of a book, then our information content will not change if the number of books fluctuates, so what matters is not their repetitive sequences but their structural properties; they can serve as bricks but not uniquely informative books. Repetitive sequences are not conserved in general, except for the rare cases when they alter exon structures and acquire “normal” functionality. Some animals and plants have genome sizes that are both much larger and much smaller than mammalian genomes (while the number of genes is approximately the same), so we can conclude that the genome size does not strongly affect organism performance. On the other hand, there should be a balance between the proliferation of these elements and some opposing force, because an unbounded multiplicative process would lead to an exponential blowup. We speculate that this balance is the independent (from usual substitutions) degree of freedom for phenotype tinkering, and its model can be developed along similar lines with the presented model. The tinkering probably affects large-scale chromatin organization and in principle is not much different from the usual mutagenesis. However, it might be useful that it is independent from it. Once the 3D nuclear organization became functionally important, some means of tinkering provided additional dimensions of variability. It seems that substitutions and small indels cannot significantly affect nuclear organization (in a direct way), so they are not well suited for this dimension of phenotype tinkering, in comparison with large-scale rearrangements and mobile elements. However, the proliferation of such elements does not immediately imply progressive evolution, an increase in complexity (which yet has to be defined formally for such elements). The balance of these elements’ activity is analogous to the mutation-selection balance of normal mutations, where we showed that the increase of complexity is not happening without special “creative” events that affect the balance (mutation rate decrease and so on), and that balance is reestablished quickly after such events.

Also, we can speculate that during replication polymerases act in different modes in repetitive and unique sequences. Otherwise, it is slightly puzzling that huge genomes of some animals and plants do not produce some kind of “replicative load”, while the channel capacity hypothesis suggests that polymerases are working hard to minimize error rates and “physiological costs”. It is known that for error correction, polymerases can use homologous sequences; we can then speculate that when such sequences are abundant the costs and/or speed of replication are significantly affected.

Another thriftiness-based (posterior) “prediction” is CpG sites. They are heavily underrepresented in mammals and some other lineages due to their hypermutagenesis. However, it is known that some functional regulatory regions contain highly conserved “CpG islands”. Either they are protected from mutagenesis by some special mechanisms or by simply purifying selection—apparently there are additional costs associated with their usage. These costs must be balanced by some benefits, and indeed they have an additional informational capacity: methylation. The same logic may apply to other over-conserved sequences such as histones.

Also, the “silent” substitutions (which do not affect protein sequence) are unlikely to be purely neutral, since organisms would capture unused informational capacity. Their suitability for calibration (null hypothesis) purposes should be carefully evaluated. Indeed, there are many reports which show their functionality potential.

In comparison with the other recently proposed explanation of Drake’s rule (Sung et al. 2012), our model does not call for additional difficult-to-define entities like “molecular refinements”, “drift barrier”, or “effective population size”. Estimates of the latter are admitted by the authors to be “fraught with difficulties”. It is not clear how to simulate that evolutionary model in silico in order to perform its validation, because genome-wide functionality and conservation is not defined. Hence, there is no specific model for selection actions, and there are many arbitrary parameters. However, a desirable feature of a “mechanistic” evolutionary model is the ability of simulation and robustness evaluation in the parameter space. Comparing Figure 1A of Sung et al. (2012) with Fig. 3 presented here, we can hypothesize that eukaryotes have lower *GI* density, on average, which is consistent with other observations (e.g., they exhibit weaker genomic conservation). Moreover, Fig. 4 demonstrates that it can be advantageous to utilize the lower density *GI* storage. The *GI* storage strategy can be affected by particular demands for optimization: e.g., viruses or bacteria might prefer compact genomes with high *GI*, for faster replication or smaller physical size, utilizing the double stranded and overlapping coding and avoiding weakly conserved regulatory non-coding DNA.

An important consequence of our reasoning is that molecular evolution on average is not about a continuous increase of total *GI*. This suggests an explanation to the naive, but still valid question of why we see “living fossils” or do not see contemporary monkeys evolving into humans continuously, (anthropocentrically) assuming the latter have higher *GI*, while on the other hand we observe an amazing morphological plasticity (e.g., dog pedigrees or Cetacean evolution). Despite being “adaptive” for a given change of environment (selection demands), evolution is not “progressive” in terms of total *GI*, since we posit that each species already has the maximum *GI* allowed by the mutation rate, which is assumed to vary slowly. That also calls for a revisiting of the popular evolutionary concept that genes operate near their best functional performance—the performance is as good as allowed by the corresponding channel capacity, balancing at the brink of “chaos and order”, so that a random mutation has a high chance of being positive, in general. The dependence of the “evolvability” on the population size is also practically a “dogma” in traditional theories, which might be a consequence of the unconstrained (“open-ended”) opportunistic “Brownian” views on evolution. However, if a population is at the *GI* limit, so that an advance in one function must be associated with “costs” to others, the role of the population size might be diminished, at least, as we showed for the maintenance mode. In this scenario, when an individual receives an advantageous mutation, its progeny will tolerate and keep more disadvantageous, new mutation-hitchhikers (and the outcomes of recombination), which will eventually nullify the effect of the initial mutation. Qualitatively, similar information “jamming” was also explored in the chapter “Conflict Resolution” in Forsdyke (2011). It seems that strong dependencies on population size in traditional models lead to some contradictions with observations, such as Lewontin’s “Paradox of Variation” (Lewontin 1974), not to mention the general trend that more evolved forms have smaller population sizes, on average. Ironically, we can draw the opposite scenario for evolvability versus population size: without immediate negative effects, random mutations will degrade the rate on average. Hence, a large population size in the long run can lead to the accumulation of variants that increase the average mutation rate, leading to degradation. The way out then is through bottlenecks: the population must be regularly refreshed by the founding of subpopulations with decreased (below the average) rates. Such subpopulations will quickly gain an advantage and overcome the main population. In a sense, it is the population genetic “ageing” analogously to a somatic ageing. In that case, the reproductive barriers, bottlenecks, and speciation events are necessities of evolution, required for the renewal and progress, rather than peculiar accidental features. We would like to remind the reader that in this model positive mutations are abundant, so there is no need in a large population size or waiting time. Periodic population “purifications” by bottlenecks prevent “smearing” of the population along the borders of the typical set—the area which, in general, is supposed to contain many “weak”, less fit alleles. Regular bottlenecks can be viewed as population-scale error correction mechanism. The latter speculation is well consistent with the concept of genome regeneration proposed by Battail (2007) as a necessary implication for the long-term reliability of genetic information transfer.

Of course, we acknowledge the role of population size in certain circumstances, for example, sudden change of environment (e.g., the addition of antibiotic to bacterial culture). However, we need to clearly separate “survivability” from “evolvability”. The same issue is with the transient hypermutagenesis, which in some cases is used to escape harsh conditions—the increased mutagenesis decreases genomic complexity in a long run (according to the model) but may serve as a survival measure; after passing through the harsh conditions, the rate is reversed to normal. Another interesting example is cancer cells, which can be qualified as being of a much simpler species: a parasite unable to survive without a host; in that case, they can afford and gain an advantage from increased mutagenesis. If we assume (for the sake of an exercise of Drake’s rule application) a cancer in equilibrium, we can speculate about the “cancer genome” size using Drake’s rule. If the (per base) mutation rate is 1,000 time higher, then the cancer functional genome size must be about 1,000 times smaller, with the rest being junk DNA for this “species”. It is tempting to establish how well such “cancer genomes” overlap in independent cancer samples.

However, when survivability is not an issue, e.g., in a gradually changing environment, without mass extinctions, we suggest that evolvability does not significantly depend on population size. The relative contributions of diverse evolutionary strategies must be further investigated; our point here is that to disentangle these issues, it is necessary to account for the evolution of genomic complexity. As such, we introduced here one possible way of accounting for total *GI* (there are likely other ways to accomplish this (Watkins 2002; Battail 2014; Adami 2004 and references in Shadrin et al. 2013)). Figure 2 illustrates the role of population size in our model. A smaller population size leads to more “drift” (including the “fixation by drift”), stochastic fluctuations of frequencies. Nevertheless, these fluctuations have a defined global average that can be revealed with a larger population size (or sufficiently long averaging). However, the population size has no impact on the total *GI*. Hence, the smaller population size is not detrimental for species complexity, as opposed to classical views where “slightly deleterious” variants are more prone to fixation by drift, leading to genomic “meltdown” and other problems. In general, it might be misleading to imagine evolution as a chain of fixation events. The model shows how evolution can proceed without any fixations, just by shifting allele frequencies, while observable fixations are mostly the consequence of drift, which might cause confusion because drift depends strongly on population size. The “drift” in our model is presented as a drift inside a huge typical set, so that for a reasonable population size the stochastic frequency fluctuations and fixations can be observed for numerous weakly conserved sites. Our model is “deterministic” in the sense that an environment is fully formalized with *GI*-profile, making the system “closed” in a sense, and thus more analyzable. Such “determinism” can be compared to the situation in thermodynamics: the trajectory of an individual molecule is unpredictable (though having certain boundaries in a phase space), however, when we average over large number of molecules we obtain some well-defined laws and distributions. Analogously, individual lineages can drift randomly (within a typical set) while the average over all possible lineages reveals the species-specific boundaries (i.e., the typical set and total *GI*). In any case, a given species must have some defined “deterministic” boundaries.

The adaptation to new selection demands then happens at the price of decreasing adaptation to other demands, a phenomenon well known to breeders (who now may attempt to select for the lower mutation rates also). For our model, this can be imagined as a reshaping of genomic *GI* profile (and corresponding phenotype) while keeping the total *GI* constant. In biological interpretations, it is the directional decrease of variability (reflected in the increase of corresponding *GI*) of one phenotypic feature (which is in demand), while increasing the variability (“loosening up”) of others. A vast amount of literature is dedicated to searching for traces of positive selection in genomes. We reason that for a complete (“physical”) picture, the evidence of (local) positive selection should be complemented with the evidence of corresponding degradation in other genomic locations. The traditional relative fitness function alone is unable to distinguish between the “reshaping” (change in *GI*-profile) and “progressive” (increase of total *GI*, decrease of mutation rate) evolution modes, because the channel capacity notion is absent in traditional models (except for the somewhat analogous “error threshold” considerations, which are presumed to be narrowly applicable in some special cases). The general properties of such “reshaping selection” (which seems to be much more frequent than the progressive mode) can be easily modeled with the suggested IT framework, to evaluate its basic features and the influences of diverse evolutionary strategies. In the case of eukaryotes, we can expect that such evolutionary plasticity resides mostly in non-coding regions with low *GI* density, since the fraction of beneficial mutations among random mutations is higher in weakly conserved regions.

## Glossary

*Equilibrium population*: a population that has evolved for an infinite time without any disruptive events.
*Genetic information* (*GI*): For a single nucleotide site (position), *GI* quantifies the bias of allele frequencies from uniformity in equilibrium population. The sum of positional *GIs* over all positions quantifies *total genetic information* and is interpretable as localization information. It serves as an estimate of genetic complexity, and we posit that it is crucial to trace this value in the course of evolution. A species with its environment can be fully represented by its genomic *GI*-profile or in the more general case by a *typical set. GI* (profile) is a species-specific property characterizing the closed system phenotype-environment; we show how to connect it with our fitness expression, which is the individual genome property.
*Typical set*: a set of all sequences which produce a species-specific phenotype. For rather long sequences, a good approximation to the total *GI* is given by the formula: −log(*TS*/*N*), where *TS* is the size of the *typical set* and *N* is the total number of possible sequences. In the case of independent positions explored here, the *typical set* can be represented with *GI*-profile (“sequence logo”).
Fitness: a degree of “typicality” of a sequence with respect to the GI-profile defined by the typical set, the likelihood of occurrence of the sequence in a typical set. From the viewpoint of the selection process occurring in our simulations, this fitness is equivalent to the fitness in its common meaning—i.e., it reflects the ability of an organism to survive and reproduce. So, we can use “fitness” in its general meaning interchangeably.
*Random genetic drift*: random changes in population allele frequencies from one generation to the next due to the finite number of individuals contributing alleles to the next generation.
*Fixation*: an allele is fixed, when its frequency is equal to one. Fixation may occur due to selection pressure or random drift.
*Channel capacity*: upper bound of the information transfer rate for which reliable (without degradation) transmission through a given noisy channel is attainable. Our central hypothesis here is that genomes of all species operate near their channel capacity determined by the mutation rate and other parameters.
